# Systematic adaptation of the Thai version of the supportive and palliative care indicators tool for low-income setting (SPICT-LIS)

**DOI:** 10.1186/s12904-021-00729-y

**Published:** 2021-02-19

**Authors:** Supakorn Sripaew, Orapan Fumaneeshoat, Thammasin Ingviya

**Affiliations:** 1 Department of Family and Preventive Medicine, Prince of Songkla University, Faculty of Medicine, Hat Yai, Songkhla, 90110 Thailand; 2 Research Center for Cancer Control, Prince of Songkla University, Hat Yai, Songkhla, 90110 Thailand; 3 Medical Data Center for Research and Innovation, Prince of Songkla University, Hat Yai, Songkhla, 90110 Thailand

## Abstract

**Background:**

Identification of patients who might benefit from palliative care among countries with different socioeconomic and medical contexts is challenging. The Supportive and Palliative Care Indicators Tool for a Low-income Setting (SPICT-LIS) was designed to help physicians identify patients in low-income setting who might benefit from palliative care. We aimed to systematically adapt and refine the SPICT-LIS for Thai general palliative care providers.

**Methods:**

We followed the WHO guidelines for translation, cross-cultural adaptation and validation of an instrument for the SPICT-LIS. Three expert panel members did the initial adaptation using forward and backward translations with pretested data. Two iterations of pretesting were conducted to test for applicability and reliability. The case vignettes which were used in the pretesting were modified hospital medical records. The pretesting was done with 30 respondents from various specialties in a community health center and 34 general palliative care providers from a regional referral hospital in the first and second iterations, respectively. To examine instrument reliability, interrater reliability and internal consistency were evaluated. Cognitive interviewing was conducted using semi-structured interviews with general practitioners (GPs) using the “think aloud strategy” and “probing questions”.

**Results:**

The adapted Thai SPICT-LIS had a total of 34 indicators which included 6 general and 28 clinical indicators. The assessment of the adapted Thai SPICT-LIS found that it provided consistent responses with good agreement among the GPs, with a Fleiss kappa coefficient of 0.93 (0.76–1.00). The administration time was 2.3–4.3 min per case. Most respondents were female. The 8 interviewed GPs said they felt that the SPICT-LIS was appropriate for use in a general setting in Thailand.

**Conclusion:**

The study found that the Thai SPICT-LIS could be an applicable, acceptable, and reliable tool for general palliative care providers in Thailand to identify patients who might benefit from palliative care.

## Background

There is a widespread need of palliative care worldwide. More than 4o million people suffer from communicable or non-communicable diseases. Patients with life-limiting illnesses suffering from a wide range of diseases may be indicated for palliative care. Increases in the ageing population in most countries are also adding to the burden of palliative care providers and creating new challenges to health policy makers. In the face of this increasing need, however, only a small number of patients who need palliative care currently have access to these services [1].

Several barriers are involved with lack of accessibility to palliative care, one of which is economic [1]. There are differences in the types and levels of care available among countries with different economic levels. Essential treatment availability may be limited by state policy [1]. For example, some studies have found that morphine was available to only 20% of patients in low- to middle-income countries, which make up approximately 80% of the global population [2]. Disease burden may vary among different economic levels and country policies. According to one epidemiological study, the burdens of communicable and non-communicable diseases and injuries were remarkably higher in South-East Asia region (SEAR) countries than in high-income countries and there was also a variety of disease contexts among the SEAR countries with different demographics and economic levels [3].

Identification of people who need palliative care has been challenging due to the presence of various disease trajectories and difficulties from unpredictable disease exacerbations [4]. In recent years, various instruments have been introduced that can help healthcare workers identify patients who might benefit from palliative care [5]. However, the applicability of such tools is influenced by various biological and socioeconomic factors, including country disease profile, economic level and language barriers [6]. The Supportive and Palliative Care Indicators Tool for Low-income setting (SPICT-LIS) is one such tool, which was developed from the original and validated version of SPICT [7] for use in Nepal. Even though Thailand has become a middle-income country in recent decades, the patterns of disease mortality in Thailand are similar to those of Nepal [3]. However, when adapting the SPICT-LIS from Nepal for use in Thailand was considered, there were concerns about issues regarding disease context and English language barriers in Thailand [8]. Therefore, this study was undertaken to revise the SPICT-LIS for use in Thailand among healthcare professionals, considering the different cultural and language factors in this country.

## Methods

### The research instrument: SPICT-LIS

The SPICT-LIS was refined from the original SPICT for use in Nepal with the cooperation of the original SPICT developers. Following the original SPICT, there are three parts in the SPICT-LIS. The first part examines six general health indicators, the second part includes ten specific life-limiting illnesses clinical indicators, and the third part is a guideline for a palliative care approach to the patient, relatives, and other professionals. The SPICT-LIS provides no definite criteria on how many positive indicators overall or how many positive indicators from the general health indicators and clinical indicators sections indicate the need for palliative care. Previous SPICT validation studies suggested that palliative care might be indicated in a SPICT with ≥2 positive general indicators and ≥ 1 positive clinical indicator [9, 10].

### Adaptation process of the instrument

**Fig. 1:**
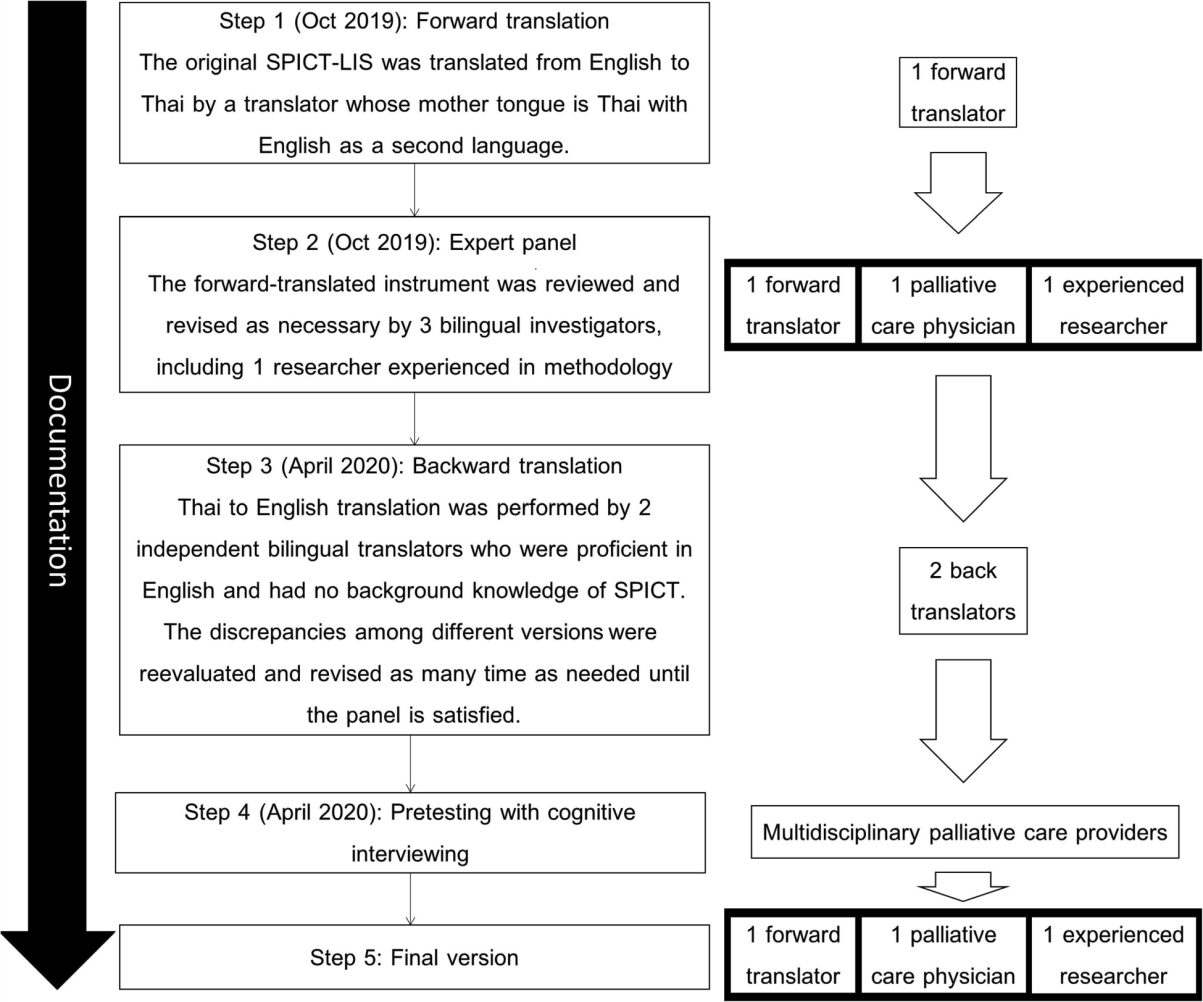
Process of adaptation of the study instrument

To translate, refine, and test the SPICT-LIS for potential use in Thailand, we followed the WHO guidelines for translation and cross-cultural adaptation of an instrument [11]: forward translation, expert panel, back translation, pre-testing and cognitive interviewing, final version, and documentation, as shown in Fig. 1.

**Fig. 2:**
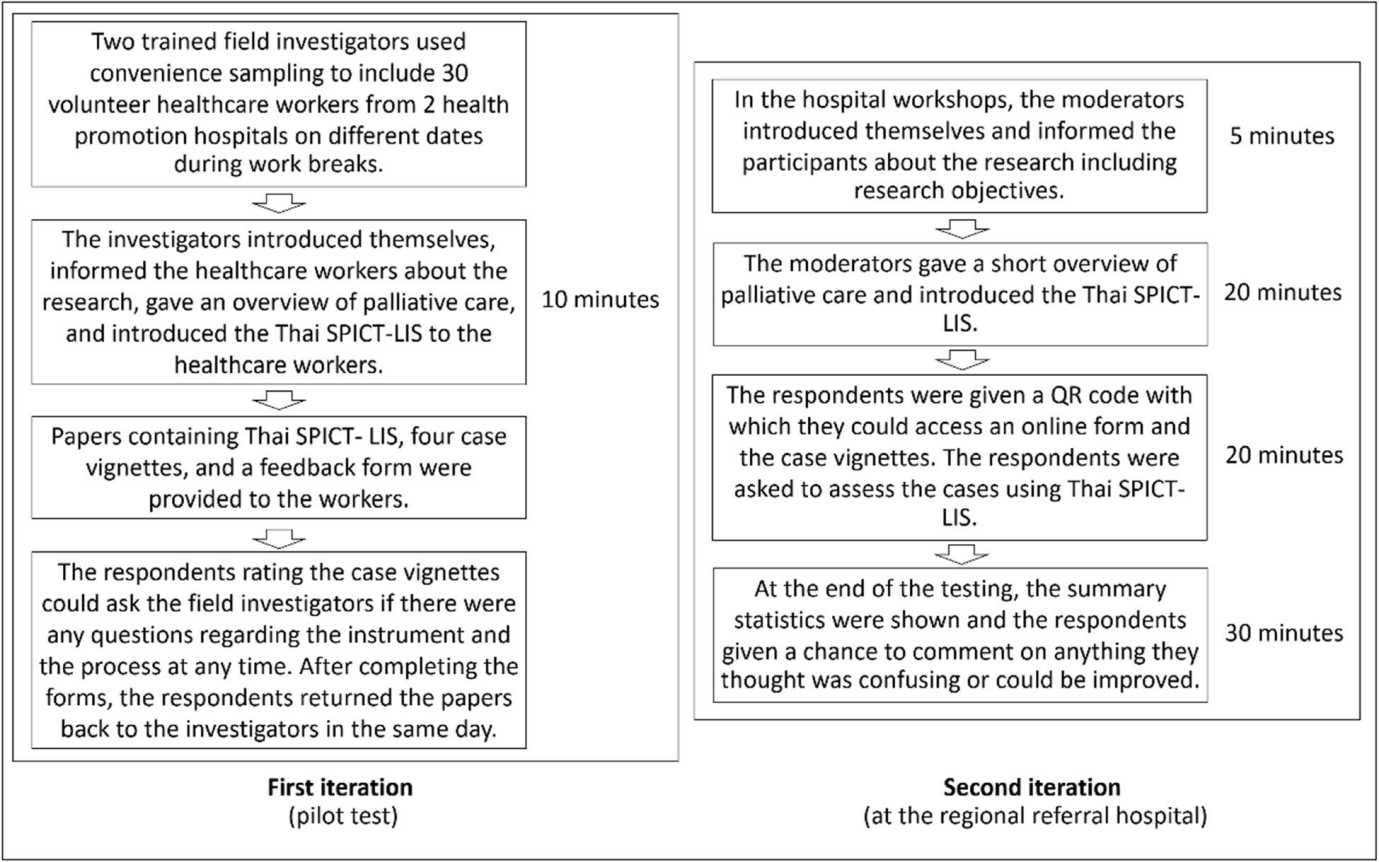
Content and steps of two pretesting groups

First step: forward translation, the original version of SPICT-LIS was translated by one independent translator whose mother tongue was Thai and second language English.

Second step: expert panel: after the Thai translation was completed, it was refined by 3 researchers. The expert panel consisted of a forward translator, palliative care expert and an experienced researcher who was proficient in English and research methodology. All words and phrases were evaluated until the panel was satisfied that the Thai translation was semantically and idiomatically equivalent to the Nepal adaptation. 

Third step: backward translation: the revised Thai translation was then back-translated to English by two independent translators who were proficient in English and had no background knowledge of the SPICT or SPICT-LIS. This translation step produced two back-translated English drafts. The discrepancies among these two back-translated versions and the original SPICT-LIS were considered in an expert panel meeting, and the Thai SPICT-LIS revised until the panel was satisfied that the two versions were essentially the same in all important aspects. 

Fourth step: pretesting: two pre-testing steps were done between January and April 2020. The overview of the process is outlined in Fig. 2. Sample size of respondents in the 2 steps was estimated based on previous guidelines regarding cross-cultural adaptation of an instrument [11, 12]. Convenience sampling of 30–40 palliative care provider with approximately 10 respondents from each discipline was applied in the study

The first step was a pilot test performed with community health promotion hospital staff to test for the instrument’s utility and reliability. Various groups of healthcare practitioners working at 2 health promotion hospitals in Songkhla, Thailand, were sampled using four case vignettes. The participants were given an overview of palliative care and instructed on how to use the instrument (http: https://www.spict.org.uk/the-spict/spict-lis/) by two trained investigators. Then, the participants were asked to complete the translated SPICT-LIS form using the sample case vignettes. Finally the participants were asked to complete a feedback form to comment on their perceptions of the test and the time required to complete it, which were then used by the expert panel to further refine the instrument’s utility and validity.

After the revisions based on the first test, a second test was then done to check the validity and reliability of the 2nd revision. This step involved 2 groups of participants with different work backgrounds from the first group in a regional referral hospital. The first group in the second testing included 23 palliative care ward nurses who were participating in a palliative care workshop held in January 2020 at Songklanagarind Hospital, Songkhla, Thailand. The nurses had voluntarily agreed to participate in the workshop. The second group included 11 general practitioners (GPs) who were participating in a monthly palliative care workshop held on April 2020 at the Department of Family and Preventive Medicine, Prince of Songkla University. Before completing the revised instrument, the participants were instructed on how to use it with a short overview lecture on palliative care and were pointed to downloadable electronic files regarding the lecture contents. However, the participants were not informed about the criteria for indicating benefit of palliative care before they completed the test procedure to avoid information bias. Only three case vignettes were used for this test of the revised instrument to assess the participants’ understanding of and the reliability of the tool. The participants anonymously accessed the test via their mobile phones using the Google Forms application during the meeting and used the revised Thai SPICT-LIS to assess the case vignettes. During the test period, the respondents could ask about words or items that they were unsure about. Semi-structured interviews with the participants were conducted after the tests were completed to discuss their perceptions towards the revised Thai SPICT-LIS and to allow them to give suggestions about improving it.

#### Development of case vignettes

Four and three case vignettes were used in the first and the second revision tests, respectively. The case vignettes were developed using patient information from medical records in the Songklanagarind Palliative Home Care service. The case vignettes used in the second revision test were further refined from feedback from the first test in terms of objective information or appropriate length and number of the case vignettes. We picked medical records of the patients who could be surrogates for patients with life-limiting illnesses and which covered all the domains assessed by the SPICT-LIS, including general health and clinical problems. The vignettes were anonymized to protect patient confidentiality. Table 1 shows short description of the case vignettes for pre-testing in the second iteration.

**Table 1:** Case vignettes for pre-testing in the second iteration

Case vignettes	Short description
1	50-year-old female, single, living with her parents at their home Stage II cervical cancer, completed chemoradiotherapy, HIV infection with good immunity and controlled viral load, CKD stage V, decided not to have dialysis Capable of doing most daily activities without any physical support Underweight, cachexic
2	59-year-old male, married, living with his wife at home Chronic alcoholism, lumbar spondylolisthesis waiting for a lumbar surgery, chronic obstructive pulmonary disease, Quit his job due to dyspnea, depends on his wife for caregiving, able to walk small distances, but stays mainly in bed or sitting in a chair
3	90-year-old male, dependent on his unmarried daughter, lives at home Stroke, vascular dementia, triple vessel disease with previous heart surgery Bedbound, on nasogastric tube, hums without meaningful words, unable to move, depends on his daughter for physical support, the daughter has chronic muscle pain and insomnia

#### Semi-structured cognitive interviews

Primary care physicians play a major role in diagnosis and patient referral in palliative care settings, and are thus considered as key users of such services. To have an acceptable level of qualitative information saturation from these key users, we purposively sampled 5–10 volunteer primary care physicians and conducted semi-structured cognitive interviews after pretesting with those physicians to test for comprehensibility and applicability of the instrument [13]. Each physician was interviewed guided by a semi-structured questionnaire using the “think aloud strategy” and “probing questions” [14] to examine their understanding of each item and to solicit any suggestions they might have for alternative terms or phrases. User perceptions and suggestions arising from the interviews were recorded and discussed with the expert panel members.

### Data collection and analysis

A mixed-methods approach combining qualitative and quantitative analyses was used in the study. The data were recorded via Google Forms and an interview record form. Quantitative data were processed and analyzed with Microsoft Excel and the R program version 3.5.1. Kuder-Richardson 20 coefficients (KR-20) were calculated to examine the internal consistency of the new instrument. Values > 0.6 were considered as showing good consistency [15]. Agreement among respondents on the need for palliative care for each vignette was estimated based on the response that indicates palliative care need, according to the SPICT-LIS criteria. Multi-rater Fleiss kappa coefficients were used to examine interrater reliability (agreement) among the respondents, with kappa coefficients between 0.61–0.80 and above 0.80 considered as substantial and almost perfect agreement, respectively [16]. Intraclass correlation (ICC) was calculated to examine the consistency of ratings among users by using the total points of general and clinical criteria rated by each user (each item valued 1 point). Values above 0.5 (moderate reliability) were considered as acceptable. Findings from the cognitive interviews were analyzed using coding frame: comprehension, recall, judgement, and response processes [17].

## Results

### Forward translation and expert panel discussion

The first revision was performed by the first expert panel meeting in October 2019 at the Department of Family and Preventive Medicine, Faculty of Medicine, Prince of Songkla University. A few corrections of the Thai translation and modifications of some parts were made in the meeting. First, the item “Cerebral malaria not responding to therapy” was considered as a criterion which was not applicable in most areas of Thailand, since Thailand had had a progressively lowering rate of overall malaria mortality and more than 80% of the country is categorized as malaria-free areas [18]. Second, the panel had a consensus that “Other infection(s) not responding to treatment and deteriorating health” was comparable to the indicator “Deteriorating and at risk of dying with other conditions or complications that are not reversible (e.g. diabetes, sickle cell disease); best available treatment will have a poor outcome.” in “Other conditions”. As a result, the criteria regarding malaria and the other infections were then removed to make the instrument suitable for use in Thailand [19]. Finally, there were 34 items left in the instrument.

### Back translation and expert panel discussion

Two back-translated versions of the SPICT-LIS were compared in the expert committee. Discrepancies between the translations were discussed and resolved differences before finally combine them into one version. The agreement among the experts resulted in:

1. A few modifications of words in 10 items, to clarify the meaning of each item in the Thai context
2. Removal of sickle cell disease from the “clinical indicators” in “other conditions” due to this disease being far less common in Thailand (sickle cell disease carrier in Thailand is less than 1% compared to the rate of approximately 17% in Nepal, the country where the SPICT-LIS was originally developed [20, 21])
3. Using English medical terms to retain the original concepts of some medical conditions regarding HIV infection and liver diseases.

### Pretesting and final version

The Thai SPICT-LIS was tested by 2 iterations. The purpose of the first iteration was to test for utility and reliability among community healthcare providers. Convenience sampling of 30 participants from community health care personnel was applied, including 7 GPs, 11 primary care nurses, 3 pharmacists, and 7 other specialties (e.g. nurse assistants). 87% of the participants were female. There were 2 comments, regarding the case vignettes and the Thai SPICT-LIS, from the pilot respondents. The first comment was that the scenario of the vignettes was not clear enough to apply the tool, and the other was about the length of the tool and number of items. The median administration time per case vignette was 3 min.

In the second iteration, the SPICT-LIS was tested with 34 participants who were asked to rate the 3 palliative care cases vignettes. 11 GPs and 23 palliative care ward nurses voluntarily participated. 55% and 100% of the GPs and nurses, respectively, were female. The characteristics of the pretest participants are shown in Table 2. There were significant differences in both age and work experience between the 2 groups. The median ages were 28 and 36 in the GP and palliative care ward nurse groups, respectively. The nurses also had a higher median of working experience compared to the doctors, 12 and 3 years, respectively. However, there was no difference in time spent for completing the instrument on each of the 3 cases which were 4.3 and 2.3 min in the doctor group and the nurse group, respectively. Eight out of eleven pretest GPs agreed to give their impressions of the test to an interviewer using a semi-structured interview.

**Table 2:** Characteristics of the second pretest participants

	General practitioners	Palliative care ward nurses	*p*-value*
Total	11	23	
Age [median (IQR)]	28.0 (26.0–29.0)	36.0 (29.0–41.0)	0.009
Work experience in years [median (IQR)]	3.0 (2.0–4.5)	12.0 (6.0–18.0)	0.004
Processing time in minutes on each case [median (IQR)]	4.3 (3.0–5.3)	2.3 (2.0–4.0)	0.051

*Computed by using Wilcoxon Rank sum test

Semi-structured interviews with the volunteer GPs were conducted following the pretest of the SPICT-LIS to explore understandings of the key SPICT-LIS users. All the responding GPs thought that the Thai SPICT-LIS could be applied in their clinical setting. There were five comments related to the clarity of the instrument regarding general criteria number two and three (patient and caregiver support needs) and some of the clinical criteria of kidney and surgical conditions that involve assessing the seriousness and deterioration of the diseases. The findings from these cognitive interviews were used to refine some of the items, resulting in adding “The patient” to the second general criterion. The final version was reviewed again by the eight doctors to be modified if there were remaining suggestions, but there were no further modifications suggested.

#### Reliability testing

For the first iteration the KR-20 results were 0.26 to 0.57 on the case vignettes, while for the second iteration with the pretest respondents, the KR-20s were 0.35–0.64. For the overall agreement among the health professionals in the second iteration, the multi-rater Fleiss kappa coefficient was 0.66 (95% CI 0.61–0.71), while the subgroup agreements were 0.93 (95% 0.76–1.00) among the 11 doctors and 0.53 (95% CI 0.44–0.60) among the 23 nurses. The intraclass correlations (ICC) for inter-rater consistency were 0.684 among the doctors and 0.523 among the nurses.

## Discussion

To systematically adapt the SPICT-LIS from the Nepal adaptation for use in Thailand, the WHO method for translation and cross-cultural adaptation of an instrument was used to modify and validate the Thai SPICT-LIS. The adaptation process was done by various experts including an experienced translator whose mother tongue was English. Medical-related problems in Thailand and length of time required to administer the instrument are important features affecting instrument application [6], so reduction and modification of items from the original Nepal instrument were considered by the panel to enhance the instrument’s applicability [19]. The validation process included several steps which resulted in the final Thai SPICT-LIS, which included 34 items, 6 general indicators and 28 clinical indicators of life-limiting illnesses. Some original English terms were kept and a few words were added to clarify some areas after the pretesting.

As we worked on the Thai SPICT-LIS, it was apparent that Thailand and Nepal had some similar economic features, but there were some health context differences that needed some instrument adaptation, e.g. burden of disease, especially communicable diseases, and health care inequities [3]. In Thailand, few tools are available in the Thai language to help clinicians identify patients who might benefit from palliative care, such as the Surprise Question (SQ), which is applicable in most palliative care settings, however, it needs to be used alongside other prognostic indicators to improve its accuracy [22]. The Palliative Performance Scale version2 (PPSv2) was adapted in 2011 for use in Thailand. The PPSv2 has five physical domains of patient performance and has been very useful in needs assessment and the other aspects of care such as patient monitoring and facilitating objective information for care team communication [23]. Thai guideline consists of the Surprise Question and various palliative care criteria based on the NHS, UK, guideline, and includes the Palliative Performance Scale and several disease-specific prognostic indicators [24]. Although there are a number of tools widely available for identifying palliative care patients, including multidimensional assessment tools [5], the application of these tools can be affected by differing socio-economic contexts leading to potential response-related biases. Also, these different contexts of disease burden might limit generalizability [6]. The SPICT-LIS is a single-page instrument adapted for a specific low-income setting, consisting of general indicators examining both physical and psychosocial domains that indicate whether a palliative care approach would be beneficial to the patient, including caregiver needs and future care plan considerations, and also includes limited-resource disease indicators in a low-income context [25], all of which are relatively close to the Thai context. This context-specific SPICT-LIS for Thailand can contribute to the national practice guideline and result in better care access.

Linguistic diversity and cultural differences can influence outcome measures [26]. The SPICT-LIS was originally developed in English and might not be readily applicable in the Thai setting [6]. In a more general Thai setting, the most common errors among Thai English users involve listening and speaking, while approximately 16% of such errors are associated with cultural differences and misunderstanding [8]. Generally when an instrument is adapted to a new language, most is translated into the new languages, but with retention of various medical terms [6]. The translation steps of this study revealed discrepancies of meanings when medical terms were translated (e.g. HIV encephalopathy). As a result, we directly imported certain English indicators to ensure the original meaning was clearly indicated.

This study evaluated the reliability of the newly adapted Thai SPICT-LIS in two aspects. Instrument consistency was evaluated in terms of internal consistency and inter-rater reliability. First, the KR20 of the instrument in this study was 0.35–0.64, indicating poor to fair values [15] while previous reports rated the reliability of the SPICT-ES from 0.70 to 0.90 [27]. This could indicate that the reliability is related to the multidimensional aspects from various life-limiting disease indicators of each version of the SPICT on different respondents with a particular set of patient scenarios and result in a variety of internal consistencies. Second, the inter-rater agreement and consistency were moderate [28], with an estimated higher value among physicians than nurses. The Kappa coefficient in our evaluation showed that the agreement among doctors was great compared to moderate values among the nurses. We suggest that the Thai SPICT-LIS could identify patients who would benefit from palliative care in terms of consistent responses among healthcare practitioners despite internal consistency variations and might result in better agreement among physicians than nurses.

To examine instrument utility, administration time and respondents’ understanding of the content were considered during the pretesting. The utility of a screening instrument must consider the degree to which the instrument can practically be used in daily practice in various pragmatic domains, for example, ease of use, specific purpose, and user acceptance [29]. One study has suggested that the administration time of a pragmatic screening tool should not exceed 10 min while theoretical relevance and user-friendly appearance were other factors enhancing acceptance among stakeholders [29]. The results from our pretesting indicated an acceptable administration time and excellent acceptance among the interviewed physicians. Based on our overall results, we believe that the Thai SPICT-LIS could help general palliative care nurses to screen patients in either outpatient or inpatient setting, and then work with the corresponding physicians to introduce palliative care approach.

### Strengths and limitations

This study had two main strengths. First, we systematically adapted the SPICT-LIS for use in Thailand using the WHO cross-cultural adaptation process with multidisciplinary professionals. The backward translation was done by two independent experienced translators from different linguistic backgrounds, helping to ensure that the back-translated versions reflected both semantic and idiomatic understandings. Second, there was a variety of healthcare specialists in our pretesting, including workers at a community health promotional hospital, and that again ensured relevant data as most palliative care services in Thailand are community-based [30]. A main limitation was that the cognitive interviewing was only restricted to physicians. Conducting cognitive interviews among nurses in the pretest would have been preferable, to check the needs for further adaptations in clinical practice. The dominance of females and the experience gap between the GPs and the nurses in our pretest respondents were observed. However, the respondent pattern approximates the actual Thai healthcare context as female dominance pattern is found overall in Thai healthcare workers and most community hospital doctors have less than 3 years of experience [31, 32].

## Conclusion

The Nepal adaptation of the English SPICT-LIS was systematically adapted and refined with multidisciplinary involvement and resulted in a reliable Thai version. The Thai SPICT-LIS can help care providers identify palliative care patients with life-limiting diseases in terms of consistent response and agreement among care providers. In our pretest situations which were designed to reflect the Thai setting, the administration time and user acceptance were good. Implementation of Thai SPICT-LIS will be able to help identify patients who might benefit from palliative care when used in addition to the national guideline and support communication process among health professionals, patients, and families.

## Data Availability

The datasets used and/or analysed during the current study are available from the corresponding author on reasonable request.

## Abbreviations

SPICT: Supportive and Palliative Care Indicators Tool
SPICT-LIS: Supportive and Palliative Care Indicators tool for Low-Income setting
SPICT-ES: Spanish version of Supportive and Palliative Care Indicators Tool
WHO: World Health Organization
GP: General practitioner
SEAR: South-East Asia region
KR20: Kuder-Richardson 20
ICC: Intraclass correlation
SQ: Surprise Question
PPSv2: Palliative Performance Scale version2
